# Immunotherapy for bilateral multiple ground glass opacities: An exploratory study for synchronous multiple primary lung cancer

**DOI:** 10.3389/fimmu.2022.1009621

**Published:** 2022-10-25

**Authors:** Lin Xu, Meiqi Shi, Siwei Wang, Ming Li, Wenda Yin, Jingyuan Zhang, Jun Zhu, Feng Jiang, Wenjia Xia, Ninglei Qiu, Zhi Zhang, Jianfeng Huang, Zhifei Ma, Fanchen Meng, Hongyu Zhu, Guozhang Dong, Jie Wang, Rong Yin

**Affiliations:** ^1^ Department of Thoracic Surgery, Jiangsu Key Laboratory of Molecular and Translational Cancer Research, Nanjing Medical University Affiliated Cancer Hospital, Jiangsu Cancer Hospital, Jiangsu Institute of Cancer Research, Nanjing, China; ^2^ Collaborative Innovation Center for Cancer Personalized Medicine, Nanjing Medical University, Nanjing, China; ^3^ Department of Oncology, Nanjing Medical University Affiliated Cancer Hospital, Jiangsu Cancer Hospital, Jiangsu Institute of Cancer Research, Nanjing, China; ^4^ Department of Pathology, Nanjing Medical University Affiliated Cancer Hospital, Jiangsu Cancer Hospital, Jiangsu Institute of Cancer Research, Nanjing, China; ^5^ Department of Radiation Oncology, Nanjing Medical University Affiliated Cancer Hospital, Jiangsu Cancer Hospital, Jiangsu Institute of Cancer Research, Nanjing, China; ^6^ Department of Science and technology, Nanjing Medical University Affiliated Cancer Hospital, Jiangsu Cancer Hospital, Jiangsu Institute of Cancer Research, Nanjing, China; ^7^ Biobank of Lung Cancer, Jiangsu Biobank of Clinical Resources, Nanjing, China

**Keywords:** immunotherapy, synchronous multiple primary lung cancer, immunogenic biomarkers, pathologic response, therapeutic indicator

## Abstract

**Background:**

Bilateral multiple ground glass opacities (GGOs) are observed in quite a part of patients with early-stage lung adenocarcinoma. For this so-called synchronous multiple primary lung cancer (sMPLC), targeting immune checkpoint is a favorable option in addition to surgical resection. The purpose of this study is to reveal the safety and efficacy of performing immune checkpoint inhibitors (ICIs) on patients with sMPLC and to explore the biomarkers of the efficacy.

**Methods:**

A total of 21 patients with sMPLC were enrolled and all included cases were pathologically confirmed adenocarcinoma after conducting surgical treatment for unilateral GGOs. ICIs of Sintilimab were then used to target programmed death 1 (200mg i.v., Q3W) for up to 10 cycles. Seven patients of them received the other surgery for contralateral GGOs, and multiomics assessments, including neoantigens, somatic mutations, and methylated loci, were further performed to investigate potential biomarkers.

**Results:**

Grade 1 or 2 treatment-related adverse events (AEs) occurred in most of the patients (12/21, 57.1%), and one subject withdrawn for grade 3 AEs. For the seven patients underwent twice surgeries, twelve and thirteen GGOs were achieved before and after the use of ICIs separately, and a favorable efficacy was observed among six lesions after immunotherapy (> 50% pathologic tumor regression). Tumor infiltration T-cell and B-cell were further shown to be associated with the biological activity of ICIs. According to mechanism-based multiomics analyses, *MUC19*- and *PCDHB5*- mutations were indicated to correlate with a favorable prognosis of sMPLC underwent immunotherapy, and our results suggested that immunogenetic mutation and associated promoter methylation could provide a quantitative explanation for the pathologic response of GGOs.

**Conclusion:**

Our study provides evidence that the use of ICIs contributed favorable efficacy and safety to patients with sMPLC. Immune infiltration and immunogenic biomarkers are revealed to be implications of performing ICIs on sMPLC. These preliminary findings exhibit the prospects in performing neoadjuvant or adjuvant immunotherapies on patients with sMPLC.

**Clinical Trial Registration:**

https://www.chictr.org.cn/showproj.aspx?proj=36878, identifier ChiCTR1900022159.

## Introduction

The detection rate of lung cancer presenting as multiple ground glass opacities (GGOs) is increasing (1). The previous research indicated that multiple GGOs are mostly multifocal adenocarcinomas, which were also identified as synchronous multiple primary lung cancer (sMPLC) (2). The natural history of sMPLC and molecular differences between isolated and multiple GGOs have not been fully examined, and the selection of appropriate intervention remains controversial (3, 4). Surgical resection, especially video-assisted thoracoscopic surgery, is considered the preferred treatment for sMPLC. However, when GGOs are scattered in different lobes in patients with more than three bilateral lesions, it remains a big challenge for thoracic surgeons (5). Therefore, an effective non-surgical treatment is urgently needed for these patients with sMPLC, especially for those who were unsuitable or unwilling to receive one more operation.

Immune checkpoint inhibitors (ICIs) have provided a major treatment advance in patients with cancer during the past decade (6). Efforts are also made to incorporate ICIs into therapy for resectable non-small cell lung cancer (NSCLC), which indicates optimal perioperative or postoperative strategy to increase overall survival. Previous studies demonstrated promising efficacy of adjuvant or neo-adjuvant ICI for early-stage NSCLC (7–10), and ICI immunotherapy has revolutionized NSCLC care in the advanced setting and can be considered as a therapeutic milestone. For the use of ICI therapy in sMPLC patients, only one case receiving three cycles of neoadjuvant pembrolizumab was reported so far (11). In this case, three solid or partial-solid nodules showed 31.8%, 12.5% and 8.3% tumor radiological shrinkage respectively, which indicates ICI therapy might be a promising strategy for extremely early-stage NSCLC patients.

Therefore, we designed a phase Ib clinical trial to observe whether immunotherapy shows efficacy on patients with sMPLC characterized with multiple GGOs. The main objective of this study is to examine the safety and feasibility of the use of Sintilimab, a PD-1 inhibitor, in residual GGOs of sMPLC patients who underwent resection of main lesion(s). Pathologic response, radiographic change, and genomic alteration after immunotherapy were revealed, and neo-biomarkers for immunotherapy were explored and identified among patients with sMPLC. Mechanistically, neoantigens were further analyzed and our results demonstrated genetic features of GGOs with immune responses. The results of this clinical trial are exploratory in immunotherapy and hypothesis generating for the treatments of sMPLC.

## Methods

### Patients

This is a single-arm phase Ib study. Eligible patients were 18 years of age or older and had bilateral GGOs [clinical stage I NSCLC, American Joint Commission on Cancer (AJCC) 8^th^ edition] that was deemed to be surgically resectable before enrollment and had an Eastern Cooperative Oncology Group performance status 0-1 (Registration number: ChiCTR1900022159, http://www.chictr.org.cn/showproj.aspx?proj=36878). From April 2019 to April 2020, 21 patients were consented on the trial and screened for eligibility (**Table 1**), and all patients were pathologically confirmed lung adenocarcinoma (LUAD) after performing surgical treatment for unilateral main lesions. These patients received Sintilimab (200mg i.v., Q3W) for up to 10 cycles after surgery. During the treatment of immunotherapy, if a grade 3 or higher adverse events (AEs) is found, the patient would be excluded. Radiological evaluations were performed at every 3 cycles within treatment period and every 3 months during follow-up. A total of seven cases finally received the other surgical resection for contralateral GGOs after the use of Sintilimab, while the others received observation only. All patients provided written informed consent before treatment and this trial adhered to all relevant ethical considerations. The present study was approved by ethical committee of Jiangsu Cancer Hospital.

**Table 1:** Patient characteristics and treatment disposition.

Patient characteristics	*n*
Median age (range)	63.9 (42–78)
Gender	
Male	9
Female	12
Smoking history	
Yes	2
No	19
ECOG PS	
0	0
1	21
Stage	
IA	20
IB	1
ICI cycles	
1	1
6	2
7	3
8	2
10	13
Adverse events	
Grade 1-2	12
Grade 3	1

### Intervention assessments

The multidisciplinary team enrolled and consented cases on the study and the clinical trial coordinator assigned consented participants to the study intervention. All the patients underwent baseline tumor staging, contrast-enhanced computed tomography (CT), positron-emission tomography CT, and magnetic resonance imaging of brain; chest CT was repeated within 7 days before surgery. Surgical resection was planned within 2 days after enrollment. Surgical resection of the unilateral GGOs and sampling mediastinal lymph nodes were performed based on surgeons’ discretion and specialty standards. The treating physicians then assigned consented participants to the immunotherapy intervention, and 6–10 cycles were determined based on patients’ tolerance. Further, changes in tumor size were evaluated according to Response Evaluation Criteria in Solid Tumors (RECIST v.1.1 (12)) by two experienced clinical radiologists. Resection of the contralateral GGOs was performed according to patients’ performance score and preference. All the patients were offered Sintilimab after surgical resection and were followed for recurrence-free and overall survival.

### Pathologic assessment

Pathologic assessment consisted of gross and histopathologic examination for the resected specimens. Patients with sMPLC were staged according to the criteria of the AJCC (8th edition) for evaluating tumor size and potentially affected lymph nodes (13). Histopathologically, the mean percentage of viable tumor cells, averaged across all reviewed tumor slides. Tumors with ≤10% of viable tumor cells were considered to have undergone major pathologic response (MPR). Partial pathologic response (pPR) was defined as >10% and<90% viable tumor cells and pathologic nonresponse (pNR) was considered as tumor with ≥90% viable tumor cells according to the previous study (14). Furthermore, pathologic responses were needed to be reviewed by two independent pathologists experienced in the evaluation of ICI response, and the average scores were used for the final analysis.

### Exome sequencing and predicting neoantigen binders

Genomic DNAs from formalin-fixed paraffin-embedded (FFPE) samples and the whole blood control samples were extracted using Qiagen QIAamp DNA FFPE Tissue Kit and DNeasy Blood and tissue kits (Qiagen, USA), respectively. Sequencing library preparation was performed with KAPA Hyper Prep Kit (KAPA Biosystems, USA). Exome capture was performed using the IDT xGen Exome Research Panel V1.0 (Integrated DNA Technologies) and sequenced using HiSeq4000 to a mean coverage depth of ~150X for the tumor FFPE samples and ~60X for the normal control. FASTQ file quality control was performed using Trimmomatic (15). Pair-end reads were aligned to the human reference genome (hg19) using Burrows-Wheeler Aligner (BWA) with default parameters, followed by PCR deduplication with Picard toolkit (http://broadinstitute.github.io/picard/). Local realignment around indels and base quality score recalibration was performed with the Genome Analysis Toolkit (v 3.4.0). Somatic single-nucleotide variants (SNVs) were identified using MuTect2. Final list of mutations was annotated using vcf2maf (https://github.com/mskcc/vcf2maf) (**Supplementary Table 2**).

Non-silent mutations were included to predict neoantigen binders. The predicted binding affinities and rank percentage scores were calculated for all peptides that bound to each of the patient’s human leukocyte antigen (HLA) alleles using netMHC (16). Using established thresholds, we predicted encode peptides that were capable of binding to at least one of the patient’s HLA class I alleles, and the results were shown in **Supplementary Table 3**. Loss of heterozygosity (LOH) events of HLA were identified by LOHHLA (17) (**Supplementary Table 4**).

### Phylogenetic analysis and principal component analysis

According to the previous study (18), mutation profiles of sMPLC per case were converted into binary format with 1 being mutated and 0 otherwise. For each patient, all validated mutations that were present in at least one tumor lesion were included. Ancestors were germ line DNA assuming with no somatic mutations. Multistate discrete-characters Wagner parsimony method in PHYLIP (Phylogeny Inference Package) was used to generate phylogenic tree. Phylogenetic trees were redrawn with relative trunk and branch lengths proportional to the number of shared and distinct mutations on the corresponding trunk or branch.

Based on the mutation profiles of binary format, principal component analysis (PCA) was performed using the R built-in function prcomp. PCA plots were generated using the first two PCs. The most intuitive distance measure is the euclidean distance between two points given by:


$$d^{2}\left(i,j\right)={\left(x_{i}-x_{j}\right)}^{2}-{\left(y_{i}-y_{j}\right)}^{2}$$

### DNA methylation detection

To construct the sequencing libraries, DNA per formalin-fixed paraffin-embedded (FFPE) sample was extracted and then treated with bisulfite. Targeted bisulfite sequencing was then performed on Illumina Hiseq platform (Illumina, San Diego, CA) using predesigned probes (SeqCap Epi CpGiant, Roche). Raw sequencing data were first demultiplexed by bck2fastq and then trimmed by Trimmomatic as part of the quality control (QC) protocol (15). The qualified reads were then mapped onto the human reference genome (hg19) using the bisulfite sequence aligner Bismark (19) after PCR duplicates removal by Picard toolkit.

The methylKit package (v 1.12.0) was used to identify differentially methylated regions (DMRs) in R (v 3.6.3). CpG clusters were tiled into 1000 bp windows with a minimum coverage ≥2 to ensure better detection of DMRs based on parameters (20). The methylation level of each DMR was calculated using the total methylated cytosines divided by the total CpGs within each window. Logistic regression was applied to calculate the methylation difference as well as the false discovery rates (FDR) between the test and control groups (**Supplementary Table 5**).

### Immunohistochemistry staining and multiplex immunofluorescence

Immunofluorescence were performed on FFPE tissue sections using an automated staining platform (Bond RX; Leica Biosystems, Buffalo Grove, IL, USA). Antibodies were validated using human tissue and cell lines as controls. Optimal antibody concentrations were determined for primary antibodies against CD8, CD20, CD56, CD68, HLA-DR, and Pan-CK. mIF using a five-colour panel (CD8, CD56, CD68, HLA-DR, and Pan-CK) and IHC using CD20 were performed on 4-mm-thick FFPE tissue sections. Slides were scanned at ×20 magnification using the Aperio ScanScope (Leica Biosystems), which were analyzed using Halo v3.2 software (Indica Labs, Albuquerque, NM, USA).

Immunohistochemistry was used to validate the infiltration of B-cells using the expression of CD20 (M075501; Dako, CA, USA). Quantification of tertiary lymphoid structure (TLS) in nodules by CD20 revealed that both TLS number and density (21) (defined as the number of TLS per unit tumor area).

### Statistical analysis

We performed pathological, genomic, and immunologic analyses on available biospecimens. Statistical analyses were performed using R (v3.5.1). For comparisons of continuous variables between groups, Mann-Whitney U tests and Kruskal-Wallis H tests were used. For comparisons of categorical variables between groups, chi-squared or Fisher’s exact tests were employed. To compare survival between groups, we used the log-rank test. All reported *P* values were two-sided. The differences were considered significant when the *P* value was<0.05. Other figures were generated using the R package ggplot2 and RColorBrewer.

## Results

### Characteristics of patients with sMPLC

**Figure 1:**
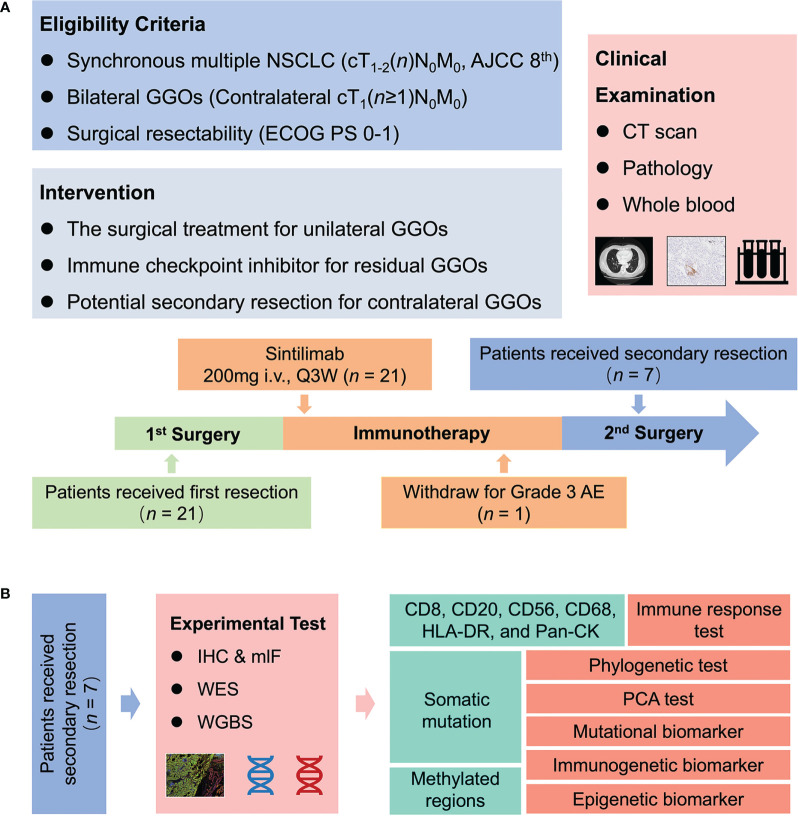
Trial schema. **(A)** Twenty-one patients with bilateral GGOs received Sintilimab (200mg i.v., Q3W) for up to 10 cycles at six weeks or later after surgical resection. During the use of Sintilimab, the radiological evaluation was performed at every three cycles within treatment period and every three months during follow-up. A total of seven cases preferred the second resection to remove the remaining resectable GGOs after 6–8 cycles of immunotherapy. **(B)** For these cases underwent twice resections, IHC staining, mIF, WES, and WGBS were performed on resected GGOs to investigate molecular features and discover neo-biomarkers. GGO, ground glass opacity; CT, computed tomography; WES, whole exome sequencing; WGBS, whole genome bisulfite sequencing; mIF, multiplex immunofluorescence; PCA, principal component analysis.

Twenty-one eligible patients received the ICIs of Sintilimab (200mg i.v., Q3W) for up to 10 cycles at six weeks or later after surgical resection (**Figure 1A**). During immunotherapy, the radiological evaluation was performed at every 3 cycles within treatment period and every 3 months during follow-up. Considering the severity of contralateral disease, a total of seven cases received the other resection to remove the remaining GGOs after 6-8 cycles of immunotherapy. To estimate therapeutic efficacy and investigate response-related biomarkers, multiomics approaches, including IHC staining, mIF, whole exome sequencing (WES), and whole genome bisulfite sequencing (WGBS), were recruited to analyze 12 and 13 resected GGOs before and after immunotherapy, respectively (**Figure 1B**).

**Figure 2:**
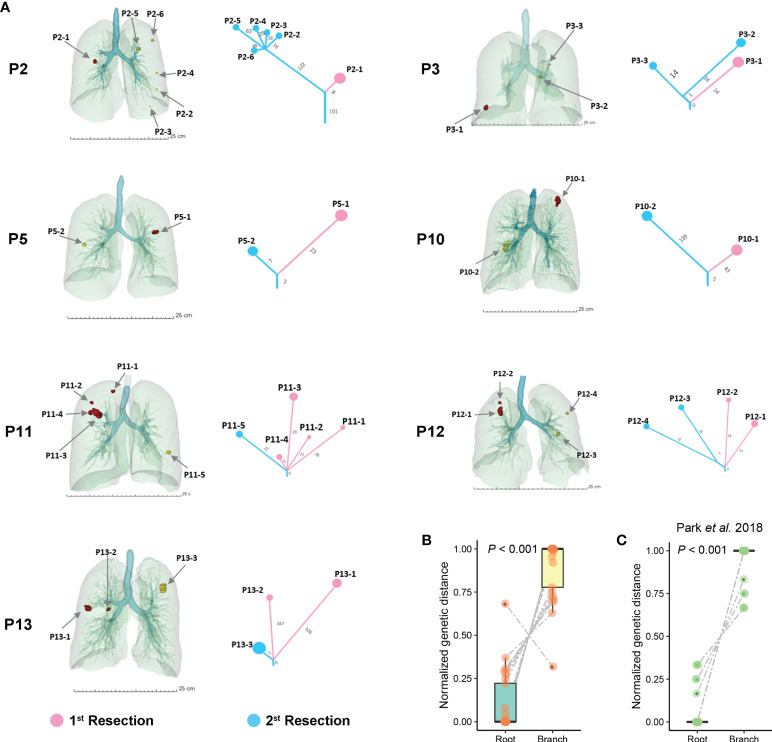
Phylogenetic trees of patients with sMPLC. **(A)** 3D locations of GGOs for patients with sMPLC. All analyzed lesions were shown before and after the use of Sintilimab. The phylogenetic relationship of multiple GGOs was demonstrated for per case. **(B)** The comparison of genetic distance between roots and branches among all included seven patients. **(C)** Phylogenetic analysis on another independent dataset of sMPLC, and the comparison between rooted and branched distance was also conducted (See Methods).

Notably, we performed phylogenetic analysis firstly to discriminate whether bilateral GGOs were multiple primary or metastatic lesions. Phylogenies were constructed for the seven cases underwent secondary resection (**Figure 2A**). The results of phylogenetic analysis indicated distinct genetic differences among multiple GGOs and demonstrated longer branch-length within seven cases (**Figure 2B**). Furthermore, we performed the phylogenetic analysis on a previous published data of sMPLC (22), which validated our results (**Figure 2C**).

### Safety and feasibility

The median treatment duration was 14.6 months (range: 3.7–19.3). One patient (1/21, 4.8%) discontinued immunotherapy owing to immune associated pneumonia (Grade 3 AE), and this patient finally recovered after the use of intravenous methylprednisolone and anti-infection treatment. In summary, grade 1 or 2 AEs occurred among most of included patients (12/21, 57.1%), and common AEs were hypothyroidism (4/21, 19%), hyperthyroidism (3/21, 14.3%), fatigue (3/21, 14.3%), and cough (3/21, 14.3%) (**Table S1**).

**Figure 3:**
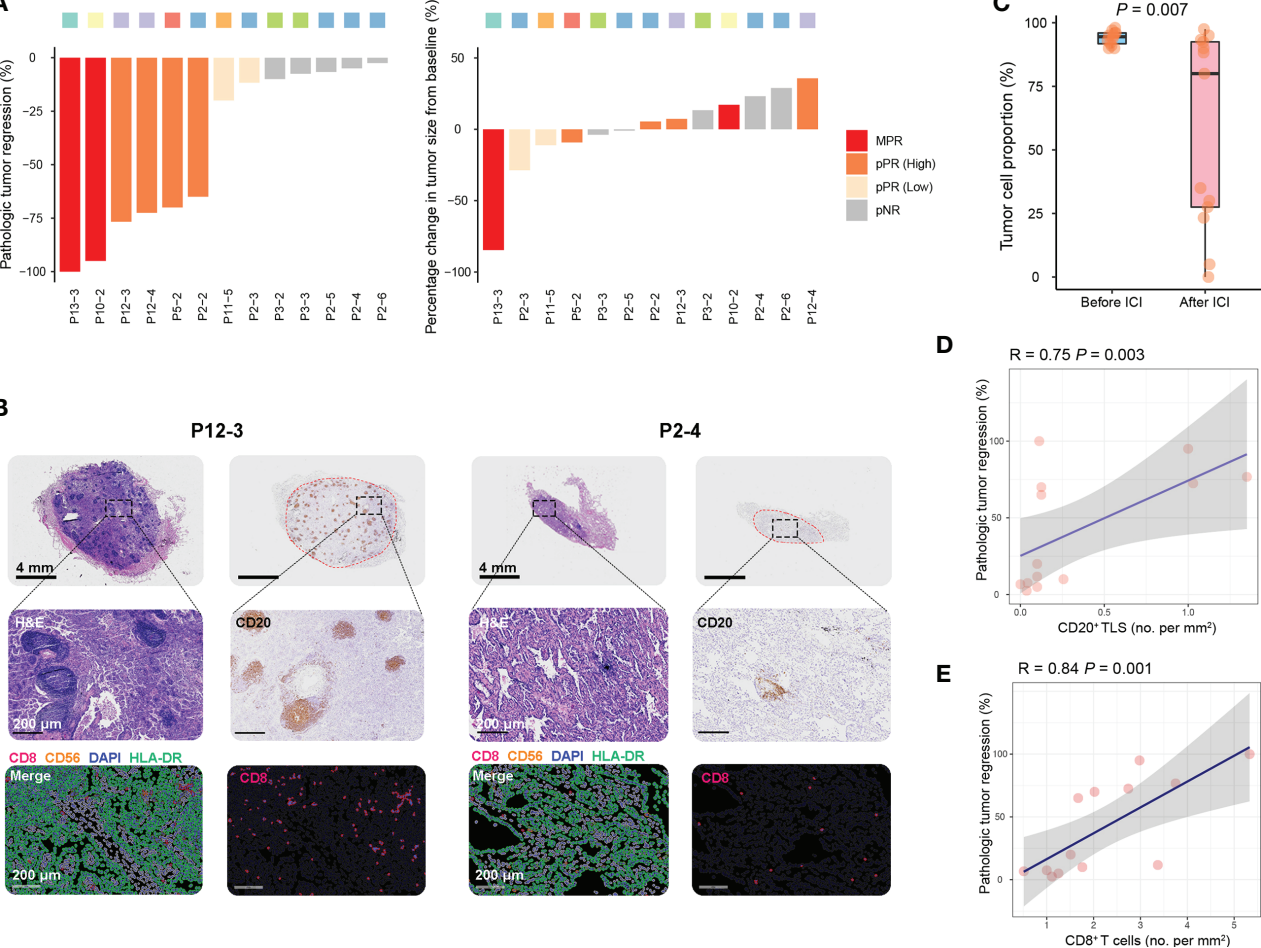
Immune responses to Sintilimab in sMPLC. **(A)** Waterfall plots of pathologic tumor regression and radiographic change in resected GGOs after the use of Sintilimab. Primary endpoint: MPR (≤10% viable tumor cells), pPR (>10% and<90% viable tumor cells), and pNR (>90% viable tumor cells). GGOs with pPR were further grouped by median value of viable tumor cell percent. **(B)** Quantification of residual viable tumor, CD20^+^ B-cells, and CD8^+^ T-cells of GGOs with favorable or poor pathologic response. **(C)** Comparison of qualifying residual viable tumor before and after the use of Sintilimab. **(D, E)** The association between CD20^+^ B-cells and CD8^+^ T-cells densities and pathologic responses among GGOs underwent immunotherapy. Quantification methods of tumor infiltrated B-cells and T-cells were shown in Methods. MPR, major pathologic response; pPR, pathologic partial response; pNR, pathologic nonresponse; ICI, immune checkpoint inhibitor.

Although the change in tumor size was moderate, the pathologic tumor regression was observed among GGOs after the use of ICIs (**Figure 3A**). For the 13 GGOs of the seven patients who received the secondary surgical resection, two lesions (2/13, 15%) of two cases achieved MPR. pPR was found in six nodules (6/13, 46.2%) including four GGOs with pPR^high^ (>10% and<50% viable tumor cells) and two pPR^low^ (>50% and<90% viable tumor cells) lesions. pNR was found in the 5 GGOs (5/13, 38.5%) of cases P2 and P3. Furthermore, we estimated the results of IHC and mIF of GGOs, and we found decreased viable tumor cells and enriched T-cells and B-cells in GGOs with pathologic regression (**Figure 3B**). Overall, the remarkably regressed tumor cells were observed among GGOs after immunotherapy (**Figure 3C**). In addition, the enriched immune cells were found to be associated with pathologic tumor regression (**Figures 3D, E**), which was consistent with the previous report (23).

### Mutational features of sMPLC underwent immunotherapy

**Figure 4:**
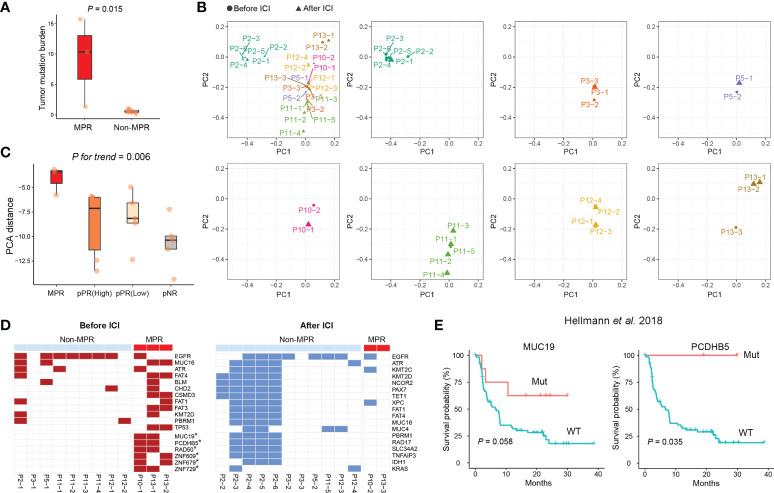
Mutational features of sMPLC before and after ICI immunotherapy. **(A)** Comparison of TMB between GGOs with and without MPR. **(B)** Genomic features of all analyzed GGOs among included patients. **(C)** Distinct genomic differences among GGOs with MPR, pPR, and pNR. **(D)** Frequent somatic mutations among GGOs with MPR before (left panel) and after (right panel) the use of ICI. **(E)** Kaplan-Meier curves using an independent cohort to estimate the prognosis of *MUC19*- and *PCDHB5*- mutated cases after immunotherapy. MPR, major pathologic response; pPR, pathologic partial response; pNR, pathologic nonresponse; ICI, immune checkpoint inhibitor.

For the case underwent MPR, we found a higher level of tumor mutation burden (TMB) among GGOs before the use of ICIs (**Figure 4A**). Intriguingly, PCA analyses showed case-specific characteristics of sMPLC, and we found that the GGOs within one patient showing distinct genomic features from other cases (**Figure 4B**). Additionally, these distinct genomic differences could be observed among cases with MPR, pPR, and pNR (**Figure 4C**, See Methods), which buttressed an anti-tumor effect of ICIs.

To identify potential response-associated mutations, we performed analyses on frequent mutations before and after the use of ICIs, respectively (**Figure 4D**). Notably, the results demonstrated that somatic mutations of *MUC19*, *PCDHB5*, *RAD50*, *ZNF609*, *ZNF679*, and *ZNF729* were enriched in GGOs from the cases with MPR before the use of ICIs. Therefore, we further estimated the association between these mutations and the efficacy of immunotherapy in a published lung cancer dataset, and the results demonstrated that *MUC19* and *PCDHB5-* mutations were related with favorable results of immunotherapy (**Figure 4E**) (24).

### Immunogenetic and epigenetic indicators for the immunotherapy in sMPLC

**Figure 5:**
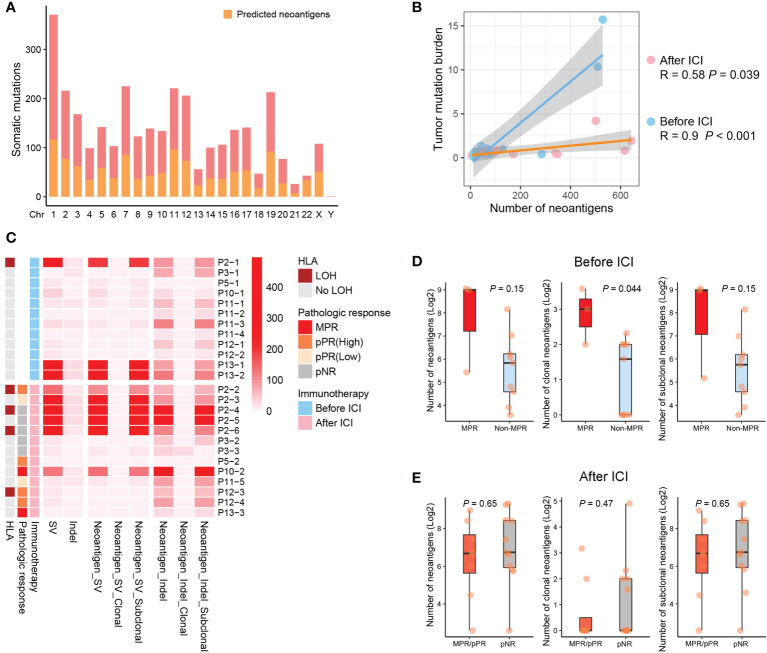
Clonal neoantigens correlates with immune responses. **(A)** Predicted neoantigens of identified somatic mutations in this study (See Methods). **(B)** Association between neoantigens and TMB for GGOs before and after ICI treatments. **(C)** For all analyzed GGOs, the clonal and subclonal neoantigen burden are suggested, as well as LOH events of HLA alleles and corresponding pathologic responses. **(D, E)** More clonal neoantigens were observed for GGOs with MPR before immunotherapy **(D)**, but no significant differences were indicated after ICI treatments **(E)**.

According to the previous study (25), we used published methods to predict neoantigens and their clonal status to investigate neoantigen depletion (16, 26) (**Figure 5A**, See Methods). Not surprisingly, the number of predicted neoantigens were associated with the level of TMB no matter before or after the use of ICIs, especially among GGOs before immunotherapy (**Figure 5B**). To be more concrete, we performed analyses on types of neoantigens, and the results showed a relatively higher level of neoantigens after the use of ICIs (**Figure 5C**). Further analyses demonstrated that the number of clonal antigens before immunotherapy was shown to correlate with pathologic response (**Figures 5D, E**), which might explain the association between TMB and treatment response.

**Figure 6:**
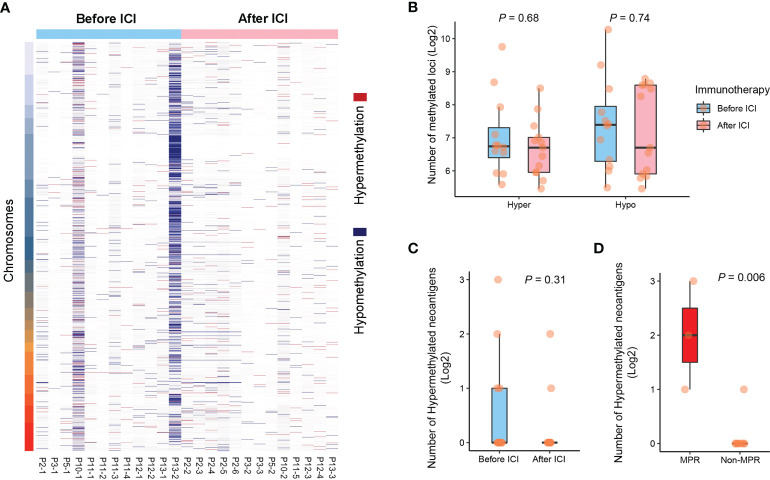
Case-specific methylation patterns explained functional neoantigens. **(A)** Hyper- and hypo-methylated loci among GGOs before and after ICI treatments. **(B)** Comparison of the number of CpGs before and after ICI treatments. **(C)** Comparison of the number of hypermethylated neoantigens before and after the use of ICIs. **(D)** The association between the number of hypermethylated neoantigens and pathologic response.

Although no statistical significance was found, we observe an increasing trend of HLA-LOH events in GGOs during immunotherapy, and these immune escape mutations amplified after immunotherapy (30.8%, 4/13) when comparing to pre-treatment ones (8.3%, 1/12) (**Figure 5C**). To investigate the biological activity of the overall immunogenetic mutations, we further detected neoantigen depletion by analyzing promoter activity, and CpG methylation patterns of promoter region were revealed using WGBS (**Figure 6A**). We observed that the number of hypermethylated and hypomethylated loci of GGOs was not affected by immunotherapy significantly (**Figure 6B**), as well as depleted neoantigens (**Figure 6C**). Intriguingly, residual repressed neoantigens were found to be enriched in GGOs with MPR (**Figure 6D**), which suggested that functional neoantigens were critical to pathologic response instead of overall antigen numbers.

## Discussion

Although multiple GGOs have been considered as the early stage of lung tumorigenesis (27), there is little understanding of pathologic response of immunotherapy in sMPLC. In this study, our results showed that the use of Sintilimab treatments on patients with sMPLC appeared favorable efficacy. Additionally, toxicities were overall manageable, with no new safety concerns compared with known safety profiles. Grade 3 or higher AEs occurred in just one enrolled case. All subjects with AEs recovered without further medication. A similar rate of treatment related AEs was also observed with patients underwent Sintilimab (28). Few studies focused on adjuvant or neoadjuvant treatments for sMPLC patients. A recent published study performed epidermal growth factor receptor tyrosine kinase inhibitors (EGFR-TKIs) on sMPLC patients after surgical operation, and a response rate of 23.9% was observed among residual GGOs (29). Besides unconfirmed pathologic response, the blind mutational status of residual GGOs would limit the extensive usage of EGFR-TKIs on sMPLC patients.

Our results demonstrated that RECIST1.1 (12), as a guideline for evaluating the efficacy of immunotherapy for solid tumors, would not be suitable for evaluating the efficacy of immunotherapy on patients with multiple GGOs. We considered the pathological evaluation of MPR, pPR, and pNR as the previous report indicated (14). Recently, Chen K et al. revealed that GGOs were associated with less immune infiltration, including lower expression of immune-related genes, down-regulation of immune pathways, lower infiltration of immune cells, and less expanded T-cell receptor (TCR) repertoire (30). However, our results shown that T-cell and B-cell associated TLSs were enriched in GGOs with favorable pathologic response (< 50% viable tumor cells), which indicated that the efficacy of immunotherapy could not be simply reflected by the immune infiltration within GGO lesions. Relatively, ICI treatments may participate in the equilibrium phase of immunoediting between cancer cells and anti-tumor immune response.

We integrated genomic features of 25 GGOs to demonstrate a landscape of included patients who received twice surgical resections. The TMB level of lesion was remarkably higher in GGOs with MPR compared with those ones without MPR. *EGFR*, *ATR*, *MUC16*, *TP53*, and *KRAS* were high frequent mutations for included cases with sMPLC. A relative low incidence of HLA LOH events (5/25, 20%) suggested that an equilibrium phase of immunoediting between tumor cells and anti-tumor immune response. A previous clinical trial of ICI treatments for metastatic tumor suggested that the high TMB level and active immune infiltration were associated with superior benefit from immunotherapy. For patients with sMPLC in the study, although we possessed a relatively low TMB level and differential immune infiltration, considerable clinical benefits were still obtained.

The major limitation of this hypothesis-generating, single-center study is the relatively small sample size. To further evaluate this hypothesis, a multicenter, randomized, phase 3 trial should be designed to generate more clinical evidence on efficacy, safety, quality of life, and translational biomarkers for patients with sMPLC. Honestly, it was difficult to monitor the pathologic response of GGOs due to pseudo progression during immunotherapy (31–33), and we did not exclude patients with driver mutations (i.e., *EGFR* and *KRAS*) or evaluate the expression level of PD-1 before the use of ICIs. This is probably the reason for a relatively low MPR rate. Despite these limitations, to our knowledge, this is the first completed trial to explore the efficacy of ICIs on patients with multiple GGOs.

## Conclusion

To our best knowledge, we provided evidence that Sintilimab contributed favorable efficacy to patients with sMPLC, which indicated a moderate rate of pathologic response and enhances tumor immune infiltrates. In addition, tumor infiltration T-cells, B-cell associated TLS, and clonal neoantigens showed potential implications to be neo-biomarkers for ICI treatments. These preliminary findings have exhibited the worthiness of the development of Sintilimab in patients with sMPLC.

## Data availability statement

The original contributions presented in the study are publicly available. This data can be found here: https://bigd.big.ac.cn accession number - PRJCA012306.

## Ethics statement

The studies involving human participants were reviewed and approved by ethical committee of Jiangsu Cancer Hospital. The patients/participants provided their written informed consent to participate in this study. Written informed consent was obtained from the individual(s) for the publication of any potentially identifiable images or data included in this article.

## Author contributions

All authors have read and approved the article. SW performed bioinformatics and statistical analysis with WY, FM, GD, WX, and ZM and drafted the manuscript with JW, WY, and RY. LX enrolled the patients with ML, FJ, MS, NQ, ZZ, JH, and RY. JYZ conducted immunohistochemistry analysis with WY and JZ. JYZ and WY contributed pathology assessment and/or samples. LX, MS, and RY proofed the manuscript. All authors contributed to the article and approved the submitted version.

## Funding

This work was supported by the National Science Foundation of China (82073235, 81872378), the Natural Science Fund for Distinguished Young Scholars of Jiangsu Province (BK20211550), and Project of Jiangsu Provincial 333 High-level Talents (BRA2020391).

## Acknowledgments

Sintilimab was provided by Innovent Biopharmaceutical Co., Ltd (Suzhou). The authors would like to thank all patients who participated in the study and the clinical personnel involved in data collection.

## Conflict of interest

The authors declare that the research was conducted in the absence of any commercial or financial relationships that could be construed as a potential conflict of interest.

## Publisher’s note

All claims expressed in this article are solely those of the authors and do not necessarily represent those of their affiliated organizations, or those of the publisher, the editors and the reviewers. Any product that may be evaluated in this article, or claim that may be made by its manufacturer, is not guaranteed or endorsed by the publisher.

## Supplementary material

Supplementary Table 2 Identified somatic mutations of GGOs before and after immunotherapy.

Supplementary Table 3 Predicted neoantigens of GGOs before and after the use of ICI.

Supplementary Table 4 Identified LOH events of HLA alleles among resected GGOs.

Supplementary Table 5 The results of DMR detection by performing WGBS.
